# A PET-CT radiomics model for immunotherapy response and prognosis prediction in patients with metastatic colorectal cancer

**DOI:** 10.3389/fonc.2025.1568755

**Published:** 2025-05-23

**Authors:** Wenbiao Chen, Peng Zhu, Yeda Chen, Guoping Sun

**Affiliations:** ^1^ Department of Gastroenterology, Shenzhen People’s Hospital, The Second Clinical Medical College, Jinan University, The First Affiliated Hospital, Southern University of Science and Technology, Shenzhen, China; ^2^ Clinical Laboratory, Shenzhen Pingshan District People’s Hospital, Pingshan General Hospital, Southern Medical University, Shenzhen, China; ^3^ Dongguan People’s Hospital Biobank, The Tenth Affiliated Hospital of Southern Medical University, Dongguan, Guangdong, China

**Keywords:** metastatic colorectal cancer, PET-CT, radiomics, TME, immunotherapy response

## Abstract

**Background:**

In recent years, radiomics, as a non-invasive method, has shown potential in predicting tumor response and prognosis by analyzing medical image data to extract high-dimensional features and reveal the heterogeneity of tumor microenvironment (TME).

**Objective:**

The aim of this study was to construct and validate a radiomic model based on PET/CT images for predicting immunotherapy response and prognosis in mCRC patients.

**Methods:**

This study included mCRC patients from multiple cohorts, including a training set (n=105), an internal validation set (n=60), a TME phenotype cohort (n=42), and an immunotherapy response cohort (n=99). High-dimensional radiomic features were extracted from PET/CT images using a deep neural network (DNN), and RNA-Seq was used to screen for features associated with TME phenotypes to construct a radiomic score (Rad-Score). At the same time, combined with immune scores (IHC staining results based on CD3 and CD8) and clinical features, a joint prediction model was developed to assess overall survival (OS) and progression-free survival (PFS). The predictive performance of the model was evaluated by area under receiver operating characteristic curve (AUC), calibration curve and decision curve analysis (DCA).

**Results:**

A radiomics signature to predict the TME phenotype was constructed in the training set and verified it in an internal validation set, with AUC of 0.855 and 0.844 respectively. In the TME phenotype external cohort, the radiomics signature can differentiate either immunopotentiation or immunosuppression tumor (AUC=0.814). In the immunotherapy response external cohort, the radiomics signature can predict response to immunotherapy (AUC=0.784). The combined nomograms can predict OS and PFS, with AUC of 0.860 and 0.875 respectively. The calibration curve and decision curve analysis (DCA) confirmed the predicting performance and clinical utility of the combined nomograms.

**Conclusion:**

In this study, a radiomic model based on PET/CT images was successfully constructed, which can effectively predict immunotherapy response and prognosis of mCRC patients. The model combines radiomic features, immune scores and clinical features, showing high prediction accuracy and clinical application value. In the future, the reliability and generalization ability of this model need to be further verified in larger prospective studies to promote its application in clinical practice.

## Introduction

Colorectal carcinoma (CRC) is the third most prevalent malignancy globally, with its mortality ranking second among all neoplasms (1, 2). Over 50% of CRC patients progress to metastatic CRC (mCRC), which is the predominant cause of mortality (3). Radical surgery is deemed the optimal therapeutic approach for mCRC patients, capable of extending the 5-year survival rate to 50% (4). Due to the complex location, large number, or large volume of metastases, a considerable portion of metastatic colorectal cancer (mCRC) patients are ineligible for surgical intervention (5). As such, the pursuit of novel and efficacious therapeutic strategies becomes imperative. The core goal of immunotherapy is to recognize and attack cancer cells by activating or boosting a patient’s own immune system. Immune checkpoint inhibitors (such as PD-1/PD-L1 inhibitors and CTLA-4 inhibitors) are the main means of immunotherapy (6).These drugs restore the anti-tumor activity of T cells by blocking immune checkpoint proteins. In recent years, immunotherapy has emerged as a potential treatment modality for tumor therapy, potentially playing a pivotal role in enhancing clinical outcomes among this cohort of initially non-operable patients (7). Despite significant advancements in immunotherapy for mCRC, nearly half of mCRC patients do not respond to immunotherapy due to the immunosuppressive tumor microenvironment (TME) (8). In addition, adverse reactions caused by overactivation or dysregulation of the immune system due to the use of immune checkpoint inhibitors, such as PD-1/PD-L1 inhibitors and CTLA-4 inhibitors, can involve multiple organ systems and range in severity from mild to life-threatening (6). Consequently, it is essential to identify patients who are responsive to immunotherapy to augment response rates and mitigate adverse events. By developing a PET-CT radiomic model to predict immunotherapy response, the aim is to identify patients who may benefit from immunotherapy, thereby reducing ineffective treatment for non-responsive patients and reducing the risk of adverse reactions.

RNA sequencing (RNA-seq) mirrors the TME immune cell infiltration status, prompting the development of various methodologies to assess immune cell density within tumor tissues, a key technique for assessing TME phenotype (9–11). Two distinct TME phenotypes have been delineated: immunoenhancement and immunosuppression. The immunoenhancement phenotype, referred to as “hot tumors”, is characterized by a high frequency of immune cell infiltration and is responsive to immunotherapy. Conversely, the immunosuppression phenotype, known as “cold tumors”, exhibits minimal immune cell infiltration, which often neutralizes the effects of immunotherapy. The transition of “cold tumors” to “hot tumors” has been demonstrated to enable a broader population to reap benefits from immunotherapy (12, 13). However, conventional approaches for TME phenotype evaluation typically necessitate invasive tissue sampling. The availability of non-invasive TME phenotype biomarkers would be advantageous. By leveraging RNA-seq data, researchers have identified the two TME phenotypes: immunoenhanced (“hot tumors”) and immunosuppressed (“cold tumors”). This foundational work paves the way for the development of non-invasive biomarkers that can predict immunotherapy response and ultimately improve patient outcomes.

Radiomics, by analyzing high-dimensional imaging data, offers extensive insights into TME phenotype (14). These radiomic characteristics not only mirror the pathological and physiological alterations within the TME but also delineate the TME phenotype, encompassing immune cell composition and infiltration dynamics (15–17). The objective of radiomics is to develop non-invasive biomarkers that reveal the correlation between radiomic features and TME phenotype. Recent preliminary radiomics findings have indicated a correlation between radiomic features and immune cell infiltration status, as well as patient prognosis and immunotherapy response prediction (18, 19). However, the application of radiomics based on PET/CT imaging to forecast immunotherapy responses in metastatic colorectal cancer (mCRC) patients remains unexplored.

In this study, a PET/CT radiomics model integrating the Rad-Score, immunoscore, and clinical parameters was established and validated across various cohorts. Rad-Score, created by the Random Forest classifier based on PET/CT radiomic features to predict tumor microenvironment (TME) phenotypes, is the core predictive tool of the study. The immune score, derived from CD3 and CD8 immunohistochemical (IHC) staining results, reflects the infiltration of immune cells within the tumor microenvironment and is a crucial indicator for evaluating TME phenotypes. Clinical features, including PET/CT SUVmax, preoperative CEA levels, and primary tumor site, are incorporated due to their correlation with immunotherapy efficacy. We propose that PET-CT radiomics can non-invasively identify microstructural differences across diverse TME phenotypes, potentially uncovering new predictors of immunotherapy response. Our goal is to classify the TME phenotype in mCRC, develop a radiomics signature for TME characterization, and prognosticate the outcomes of mCRC patients undergoing immunotherapy. Furthermore, we suggest that nomograms incorporating Rad-Score, immunoscore, and clinical parameters may offer a robust prediction of overall survival (OS) and progression-free survival (PFS) in mCRC patients. Together, these measures form a comprehensive predictive model designed to assess immunotherapy response and prognosis in patients with metastatic colorectal cancer (mCRC).

## Methods

### Study design

**Figure 1:**
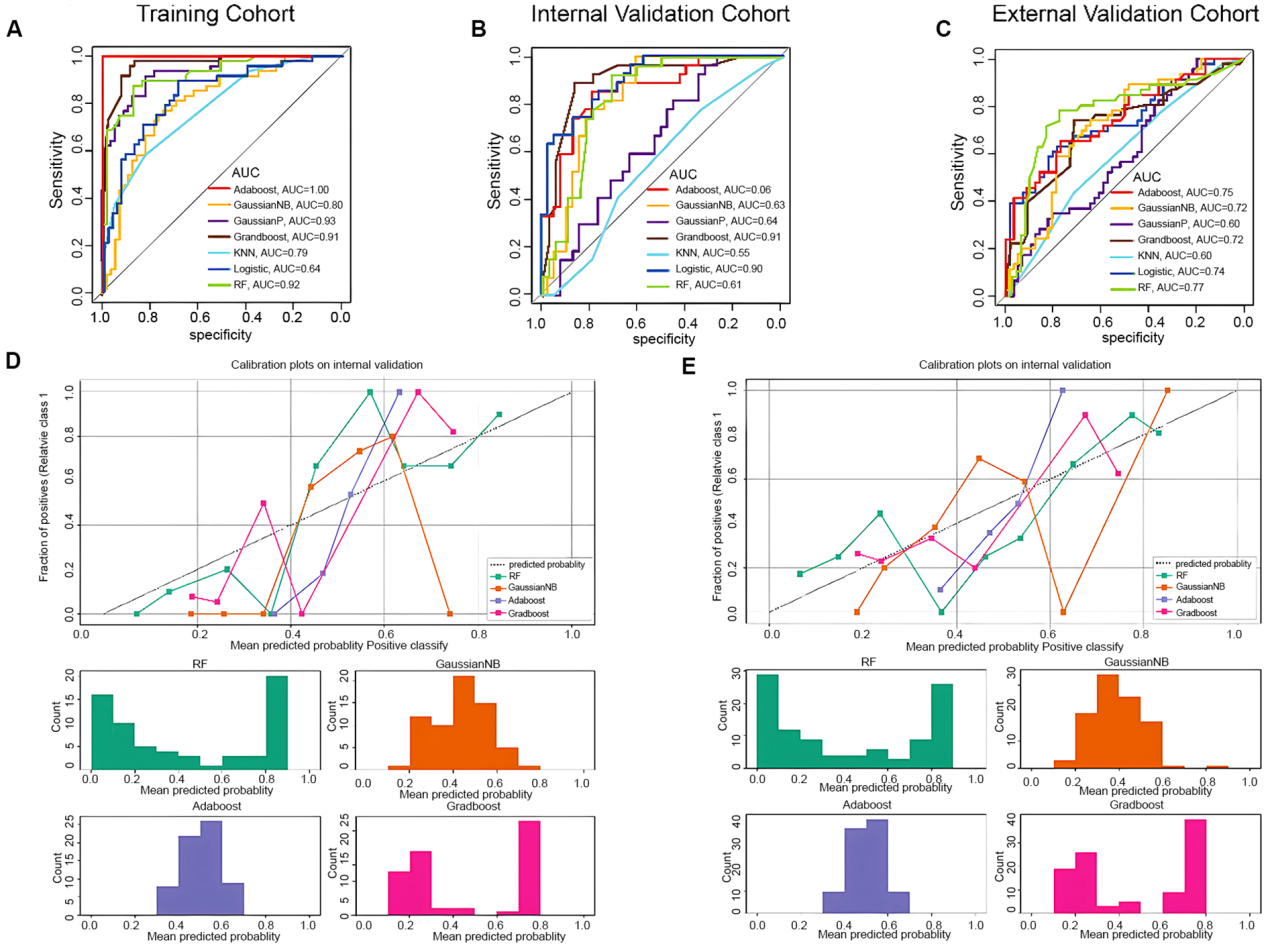
Classification algorithm comparison. AUC of different algorithms (Adaboost, GaussianNB, GaussianP, Grandboost, KNN, Logistic model, Random Forest-DERBY model) on the training **(A)**, internal validation **(B)**, and external validation cohort **(C)**. Probability Calibration analysis in internal validation **(D)** and external validation cohort **(E)**. As **(A–C)** shows, the RF, Adaboost, GaussianNB and Gradboost models yields comparable AUC scores. It can be seen from **(D, E)**, among these 4 competing algorithms, Adaboost and Gradboost models show poor calibration in, while GaussianNB tend to output probabilities close to 0 or 1. Abbreviation: KNN, K-Nearest Neighbor; RF, Random Forest.

In this multi-cohort study, retrospective RNA-Seq and radiomics analysis was performed in four distinct cohorts of mCRC patients (**Figure 1**). In order to improve the reproducibility of the study, we followed the following clear inclusion and exclusion criteria when selecting patients. Inclusion criteria: mCRC patients between the ages of 18 and 75 years; Patients must have complete PET-CT and RNA-seq data; Patients must agree to participate in the study and sign an informed consent form. Exclusion criteria: Patients with other active malignancies; Patients with severe cardiac, liver and renal dysfunction; Patients unable to complete a baseline PET-CT scan.

According to the inclusion criteria, a total of 306 patient samples were collected from Shenzhen People’s Hospital, the Tenth Affiliated Hospital of Southern Medical University and Shenzhen Pingshan District People’s Hospital, including 105 in the training set, 60 in the internal validation set, and 141 in the external validation set (**Supplementary Figure S1**). The external validation set was divided into TME phenotype group (N=42) and immunotherapy response group (N=99). The training set for mCRC patients provided PET-CT and RNA-seq data. Internal validation sets were used to confirm agreement between radiomic features and TME phenotypes. In addition, we divided the TME phenotypic cohorts into immunoenhanced and immunosuppressed groups by immunohistochemical (IHC) staining of CD3 and CD8 outcomes (20, 21). which was used to determine the correspondence between radiomic features and TME phenotypes. The immunotherapy response group was used to predict the immunotherapy response. The clinical characteristics of all patients are shown in **Table 1**. Clinical features were screened using the LASSO regression, and PET/CT SUVmax, metabolic tumor volume, pathology type and CA125 level were selected (**Supplementary Figures S2**, **S3**).

**Table 1:** Study cohort and patient characteristics.

Characteristic	Training Set (n=105)	Internal Validation Sett (n=60)	External Validation Set 1 (n=42)	External Validation Set 2 (n=99)
Sex				
Female	32 (31.4)	22 (36.7)	10 (23.8)	32 (35.6)
Male	73 (68.6)	38 (63.3)	32 (76.2)	67 (64.4)
Age, years				
Median (range)	58 (30-75)	58 (31-81)	61 (29-72)	60 (29-78)
	22 (21.4)	14 (21.5)	10 (27.0)	34 (33.3)
No. of metastases				
	10 (9.8)	11 (18.3)	6 (14.3)	25 (25.3)
≥3	95 (90.2)	49 (81.7)	36 (85.7)	74 (74.7)
Maximum size of metastases, cm				
	69 (67.6)	41 (68.3)	23 (54.8)	69 (69.7)
≥5	36 (32.4)	19 (31.7)	19 (45.2)	30 (30.3)
Immunoscore, mean ± SD				
CD3	11.9 ± 7.8	12.3 ± 8.1	11.1 ± 5.2	12.3 ± 5.0
CD8	11.8 ± 5.2	11.2 ± 3.7	12.9 ± 4.3	11.6 ± 8.6
Overall response				
CR	0 (0.0)	0 (0.0)	0 (0.0)	0 (0.0)
PR	49 (48.0)	27 (45.0)	11 (26.2)	46 (46.5)
SD	38 (37.3)	23 (38.3)	13 (31.0)	41 (41.4)
PD	16 (15.7)	15 (25.0)	13 (31.0)	15 (15.2)
ORR (CR plus PR)	49 (48.0)	27 (45.0)	11 (26.2)	46 (46.5)
DCR (CR plus PR plus SD)	87 (82.8)	50 (83.3)	24 (57.1)	87 (87.9)
PFS, years				
Median	7.5	8.5	5.2	8.0
(95% CI)	6.7 to 9.4	7.3 to 11.0	3.8 to 6.0	6.9 to 9.3
OS, years				
Median	20.7	22.8	15.5	22.0
(95% CI)	18.6 to 26.0	15.8 to 25.7	13.5 to 19.8	18.7 to 28.1
Surgery for metastases				
Resection rate	24 (23.5)	15 (25.0)	3 (7.1)	20 (20.2)
Actual R0 resection rate	23 (22.5)	15 (25.0)	3 (7.1)	18 (18.2)

Data presented % unless otherwise indicated.

CR, complete response; DCR, disease control rate; DFS, disease-free survival; NORR, objective response rate; OS, overall survival; PD, progressive disease; PFS, progression-free survival.

### Radiomics score

**Figure 2:**
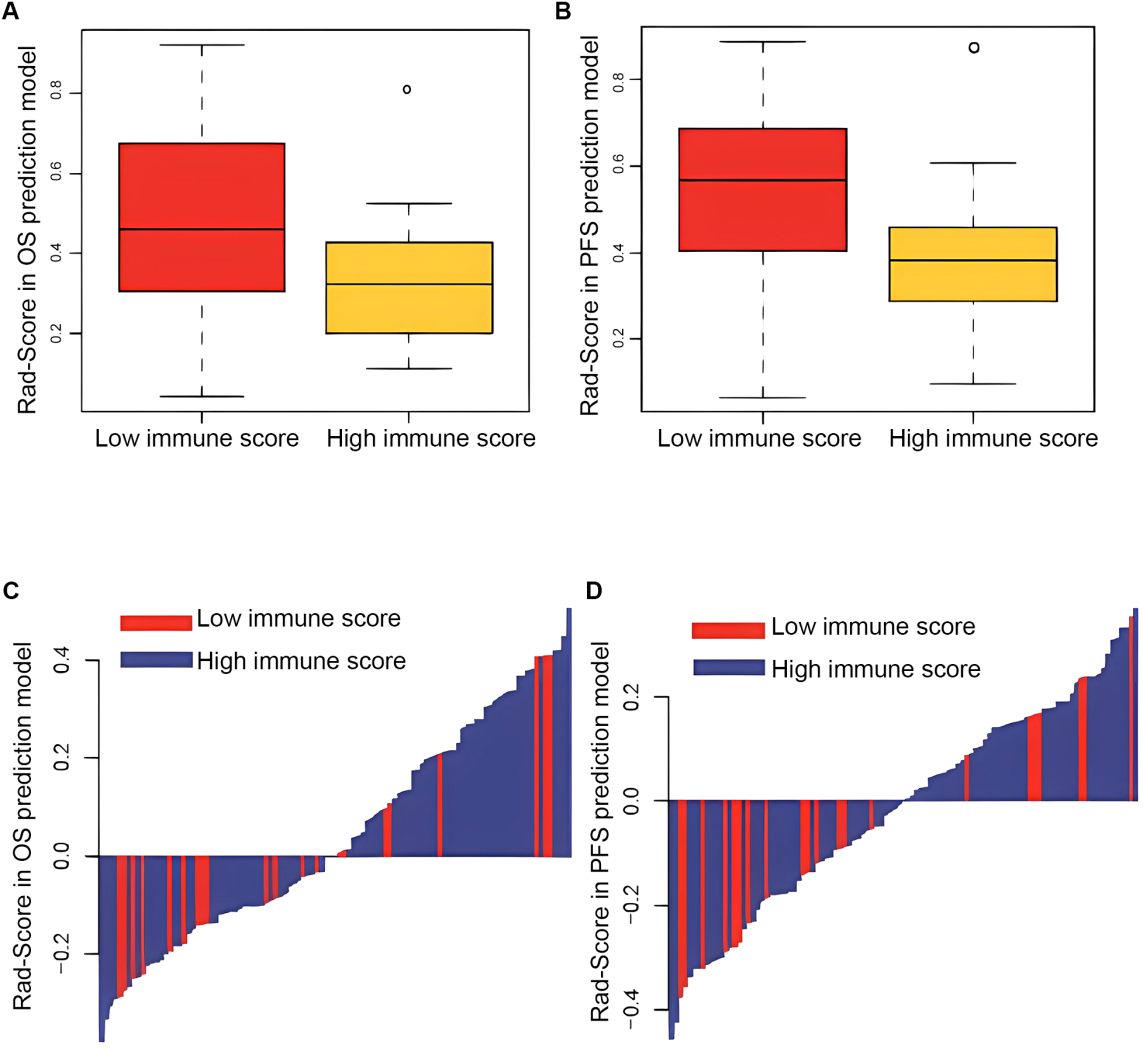
Comparison of radiomics score of OS **(A)** and DFS **(B)** prediction model between patients in low and high immune score groups. Distribution of radiomics score of OS **(C)** and DFS **(D)** prediction model and immune status.

18F-FDG PET/CT scans were performed using a uMI510/uMI780 scanner (United Imaging Healthcare, Shanghai, China) as previously described (22, 23). Three dimensions images were acquired after the 18F-FDG injection. The PET images were reconstructed and segmented using a uWS-MI workstation (United Imaging Healthcare, Shanghai, China). The regions of interest (ROI) of PET images were manually enclosed. The ROI of the CT images were delineated according to the corresponding ROI in the PET images. The CT and PET radiomics features were extracted in parallel using the deep neural networks (DNNs). These features reflect the metabolic characteristics of the tumor (from PET images) and anatomical characteristics (from CT images). After comparing several commonly used classification algorithms, the random forest classifier was used to identify radiomics features and create a predictive radiomics score (Rad-Score) (**Figure 2**). The Rad-Score was combined with immune scores and clinical features to construct the final prediction model. The predictive performance of the model is then evaluated through internal and external validation sets.

In the immunotherapy response cohort, patients were categorized into high or low risk groups based on the median value of the Rad-Score. Clinical response was assessed six months from the start of immunotherapy, including complete response (CR), partial response (PR), stable disease (SD), and progressive disease (PD). Progression-free survival (PFS) and overall survival (OS) was calculated as the period between the start of immunotherapy and the date of the last follow-up.

### Abundance of TME cell subsets

The gene expression matrix was generated from the collected tumor tissues by RNA-Sequence (RNA Seq) assay, and the presence and abundance of cell subsets were calculated by K-cluster analysis.

Cells label genes in the following way: CD3 labels T cells, CD8 labels cytotoxic T cells, CD4 labels helper T cells, CD68 labels macrophages, and CD20 labels B cells. The expression levels of these marker genes can reflect the presence and abundance of the corresponding cell subsets.

Cells label genes in the following way: CD3 labels T cells, CD8 labels cytotoxic T cells, CD4 labels helper T cells, CD68 labels macrophages, and CD20 labels B cells. The expression levels of these marker genes can reflect the presence and abundance of the corresponding cell subsets.

### Immunoscore

**Figure 3:**
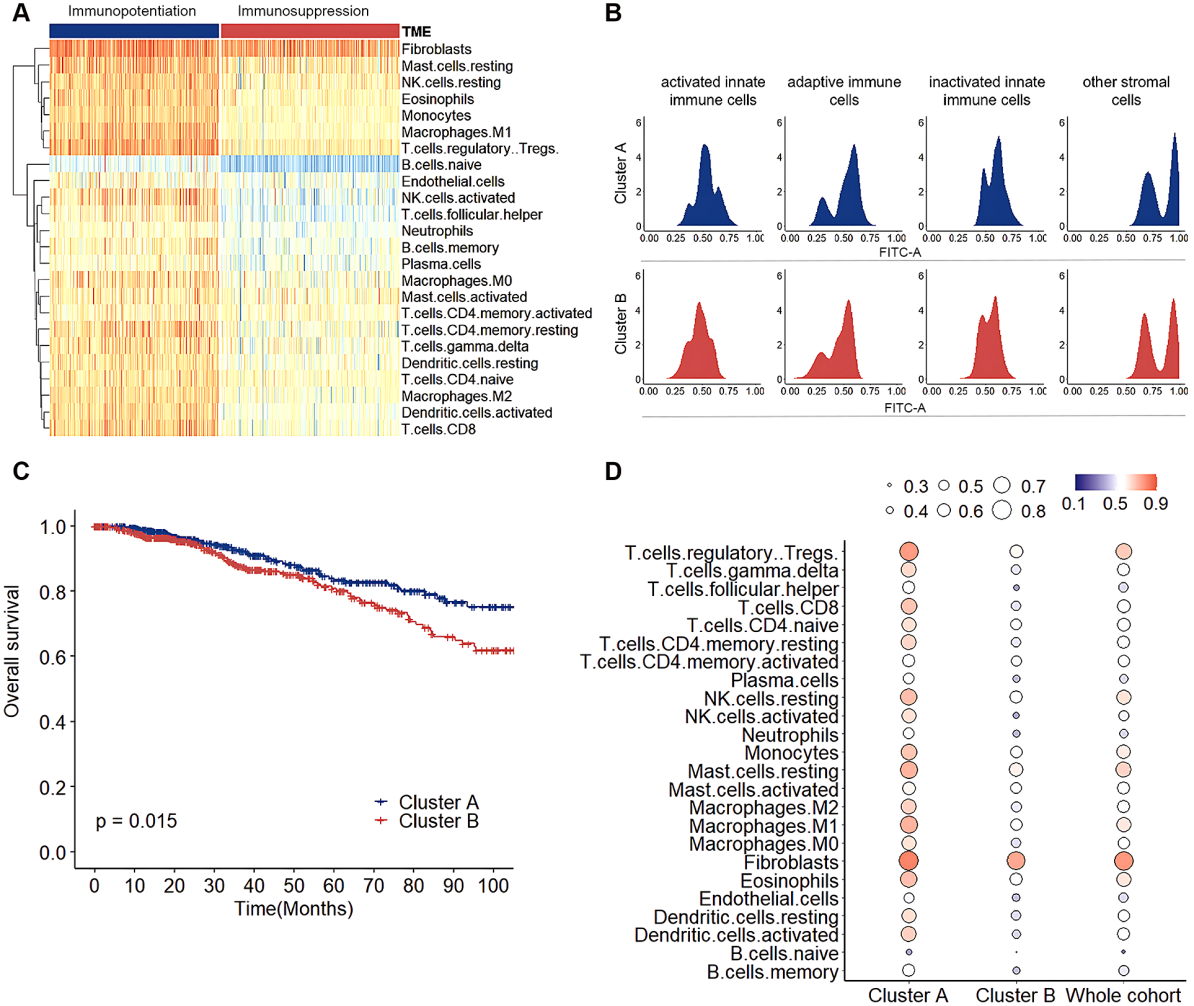
Landscape and prognostic significance of TME phenotypes in mCRC. **(A)** K-means clustering of TME phenotypes in mCRC demonstrating abundance of 24 TME cell subpopulations. **(B)** Distribution of characteristic scores of four cell subpopulations in two clusters. **(C)** Kaplan-Meier curves of OS between Cluster A and B. **(D)** Measurement of the prognostic value of each cell subgroup by a univariate Cox proportional hazards model for OS in the whole cohort, as well as Cluster A and B.

Two protein markers (CD3 and CD8) were selected to represent the TME phenotype. CD3 tags T cells, reflecting the overall infiltration of T cells in the tumor microenvironment. CD8 labeled cytotoxic T cells (CTLs) reflect the infiltration of killer immune cells in the tumor microenvironment. By the density of these two markers, TME phenotypes can be divided into: immunoenhanced (“ hot tumors “): High density of CD3 and CD8 positive cells indicates an abundant infiltration of immune cells in the tumor microenvironment and a good response to immunotherapy. Immunosuppressed type (“ cold tumor “): Low density of CD3 and CD8 positive cells indicates less infiltration of immune cells in the tumor microenvironment and poor response to immunotherapy. We obtained immune scores from the results of CD3 and CD8 immunohistochemical staining. CD3 and CD8 stained slides were scanned using a full-film scanner (Leica Microsystems, Wetzlar, Germany) under 40x magnification. The density of CD3^+^ and CD8^+^ T cells in the center of the tumor and in the infiltrating margin were measured by HALO (Indica Labs, USA). Average density ≥75% is considered “high” density. Based on the density of CD3 and CD8 positive cells in the center of the tumor and at the edge of the invasion, the immune score was assigned on a scale of 0 to 4:0 indicates that the density of both cell types is low in both areas; 4 indicates a high density of both cell types in both regions. A score of 0–2 belongs to the low immune rating group, and a score of 3–4 belongs to the high immune rating group. The higher the immune score, the richer the infiltration of immune cells in the tumor microenvironment, and the higher the possibility of immunoenhanced type. Correlation of CD3 and CD8 IHC scores and Kaplan-Meier (KM) survival analyses confirmed that these tumor-related features were associated with immunotherapy efficacy (**Figure 3**).

### Nomogram

Independent prognostic factors were identified using multi-variable Cox regression analysis. Then, two nomograms, combing Rad-Score, immunoscore and clinical features, were created to predict overall survival (OS) and Progression Free Survival (PFS) in mCRC patients. When using nomograms, it can be interpreted by adding the weighted score for each variable indicated at the top of scale. The total score can be converted into the prediction of the probability of death and recurrence or metastasis for a patient in the lowest scale. A higher total score was associated with worse OS or PFS. Decision Curve Analysis (DCA) assesses the clinical decision utility of Nomogram by comparing the net benefit of treatment under different thresholds. The DCA curve shows the net health benefit of using Nomogram for clinical decision making compared to not using Nomogram at different thresholds.

### Assessment of performance

To evaluate the performance of Rad-Score, the area under the receiver operator characteristic curve (AUC) and other metrics (accuracy, sensitivity, specificity, positive predictive value and negative predictive value) were used to determine whether Rad-Score could categorize mCRC patients into two TME phenotypes based on the abundance of TME cell infiltration.

To assess the association between the high-risk or low-risk prediction scores generated by nomogram models and prognosis, receiver operator characteristic curve (ROC) analysis, Kaplan-Meier (KM) survival analysis, and Cox proportional risk regression model were used. The calibration curve assessed the accuracy of the combined nomograms for predicting OS and PFS. Decision curve analysis (DCA) was used to assess the clinical decision utility of the combined nomograms.

### Statistical analyses

All processing and analysis steps were conducted in Python 3.8 and the R 4.0.2 software. Python 3.8 following libraries include NumPy 1.19.2, Pandas 1.1.3, Scikit-learn 0.23.2 and TensorFlow 2.3.1.R 4.0.2, which contains the following packages survival 3.1–8 and rms 6.1-0.

## Results

### TME phenotypes in mCRC

**Figure 4:**
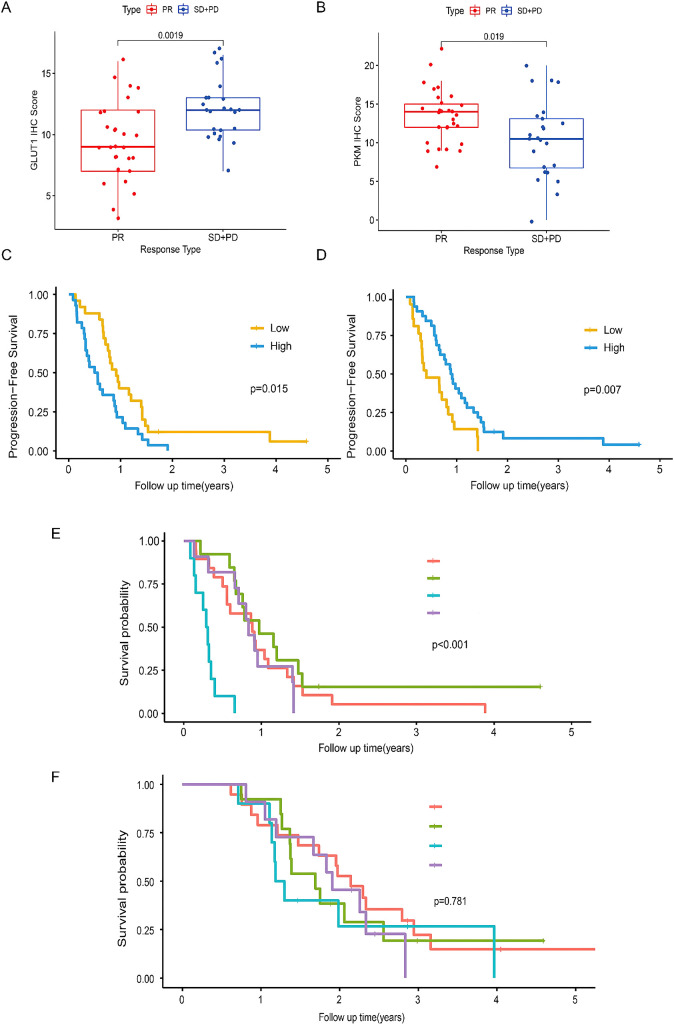
Correlation and Kaplan-Meier survival analysis of CD3 and CD8 in histology cohort. **(A, B)**. Correlation analysis of CD3 and CD8 IHC score with immunotherapy efficacy. **(C, D)** Kaplan-Meier estimates of PFS stratified by the level of CD3 or CD8 expression. **(E, F)** Kaplan-Meier estimates of PFS and OS stratified by the level of CD3 and CD8 expression. The statical analysis of IHC score was done with z-test.The statical analysis of PFS and OS was done with log-rank test. Red line, CD3 high and CD8 high; green line, CD3 high and CD8 low; blue line, CD3 low and CD8 high; purple line, CD3 low and CD8 low. Abbreviations: IHC - Immunohistochemistry.

RNA-Seq was used for quantitative analysis of the expression levels of all transcripts in a cell to reflect cell type and functional status. A total of 24 TME cell subpopulations comprehensively defined the TME phenotype (24, 25). The abundance of 24 TME cell subpopulations was measured in each sample. We then performed k-means clustering with the TME phenotype. The TME phenotype of mCRC patients was divided into two heterogeneous groups: the “immunopotentiation” group (“hot tumor”) with a relatively large infiltration of immune cells, and the “immunosuppression” group (“cold tumor”) with a low TME cell infiltration (**Figure 4**). The infiltration of immune cells was much more pronounced in the “immunopotentiation” group, while other stromal cells were much more pronounced in the “immunosuppression” group (**Figure 4**). Given the importance of the TME for the prognosis of mCRC patients, we further investigated the prognostic significance of two TME phenotypes. Through K-means clustering, we found that the TME phenotype of mCRC patients can be divided into two different clusters: immunoenhanced type (Cluster A): abundant immune cell infiltration and better prognosis; Immunosuppressive type (Cluster B): Less infiltration of immune cells and poor prognosis. Kaplan-Meier survival analysis was used to verify the difference in prognosis between the two clusters, and it was found that the overall survival (OS) of the immunoenhanced Cluster A was significantly better than that of the immunosuppressed Cluster B(p<0.05) (**Figure 4**). Better prognosis was achieved in in the “immunopotentiation” group with a larger degree of immune cell infiltration, as well as in the “immunosuppression” group and in the entire cohort (**Figure 4**).

### Radiomics signature for the prediction of the TME phenotype

**Figure 5:**
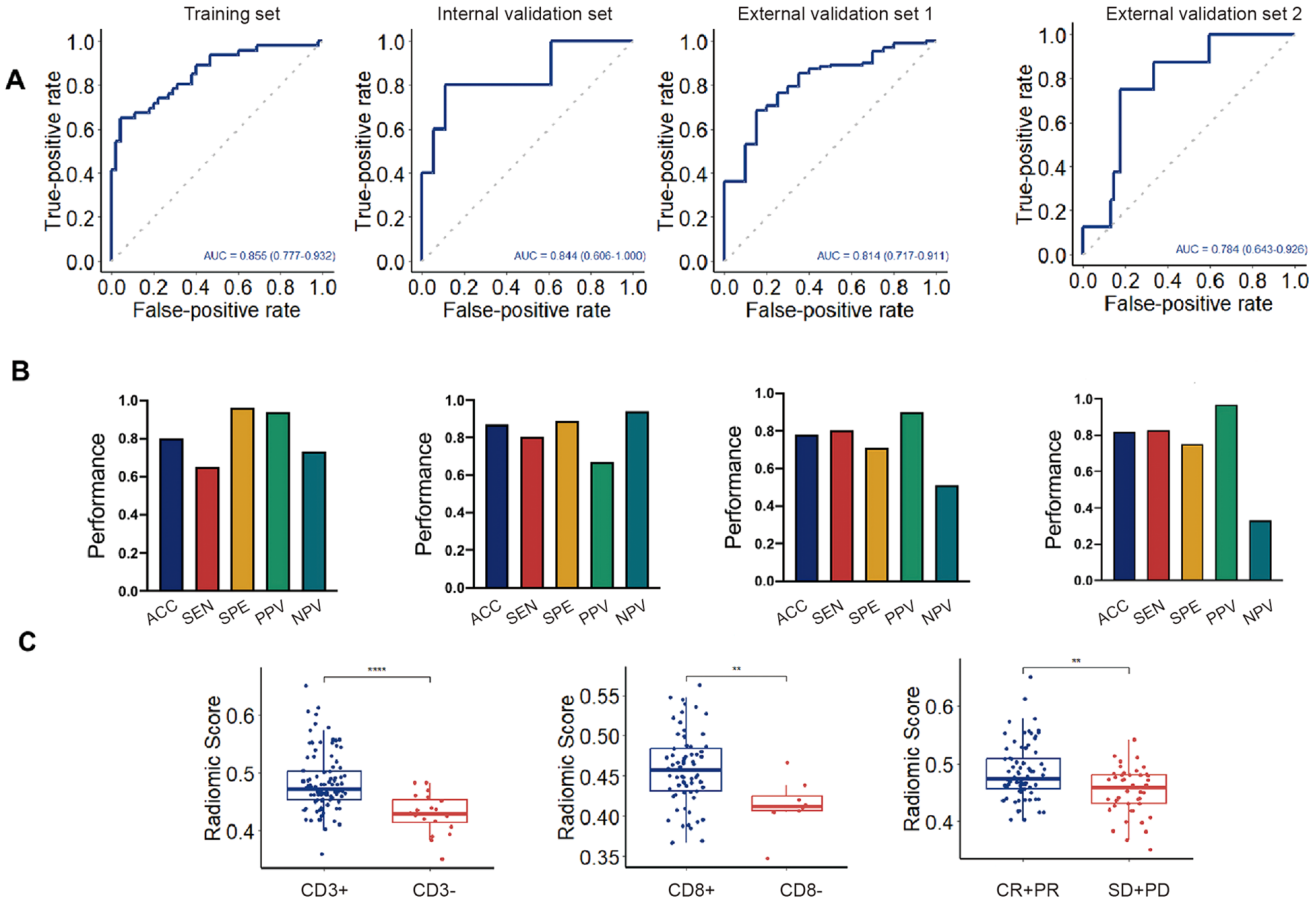
Performance of radiomics signature in the training and validation sets. **(A)**. Diagnostic efficacy of radiomics signature in the training set and three independent validation sets. **(B)**. Evaluation metrics of radiomics signature in the training set and three independent validation sets. **(C)**. Comparison of rad scores for different CD3, CD8 expression, and immunotherapy responses, respectively. ****, p<0.0001. **, p<0.01; ACC, accuracy, SEN, sensitivity, SEP, specificity, PPV, positive predictive value, NPV, negative predictive value.

A random forest classifier was used to create Rad-Score to predict the TME phenotype in the training set (AUC=0.855), the internal validation set (AUC= 0.844), and the external validation set 1 (AUC=0.814). Rad-Score could differentiate responders (CR and PR) and non-responders (SD and PD) of immunotherapy, the external validation set 2 (AUC=0.784) (**Figure 5**). Other metrics (sensitivity, specificity, positive predictive value and negative predictive value) all suggested that Rad-Score had excellent predictive performance (**Figure 5**). In addition, Rad-Score was significantly higher in the CD3-high group than the CD3-low group (p<0.01) (**Figure 5**). Similarly, Rad-Score was significantly higher in the CD8-high group than the CD8-low group (p<0.01). Responders with CR and PR had higher Rad-Score than non-responders with SD and PD (p<0.01). Thus, the above results verified the reliable predicting performance of Rad-Score for the TME phenotype.

### Prognostic value of Rad-Score and immunoscore

**Figure 6:**
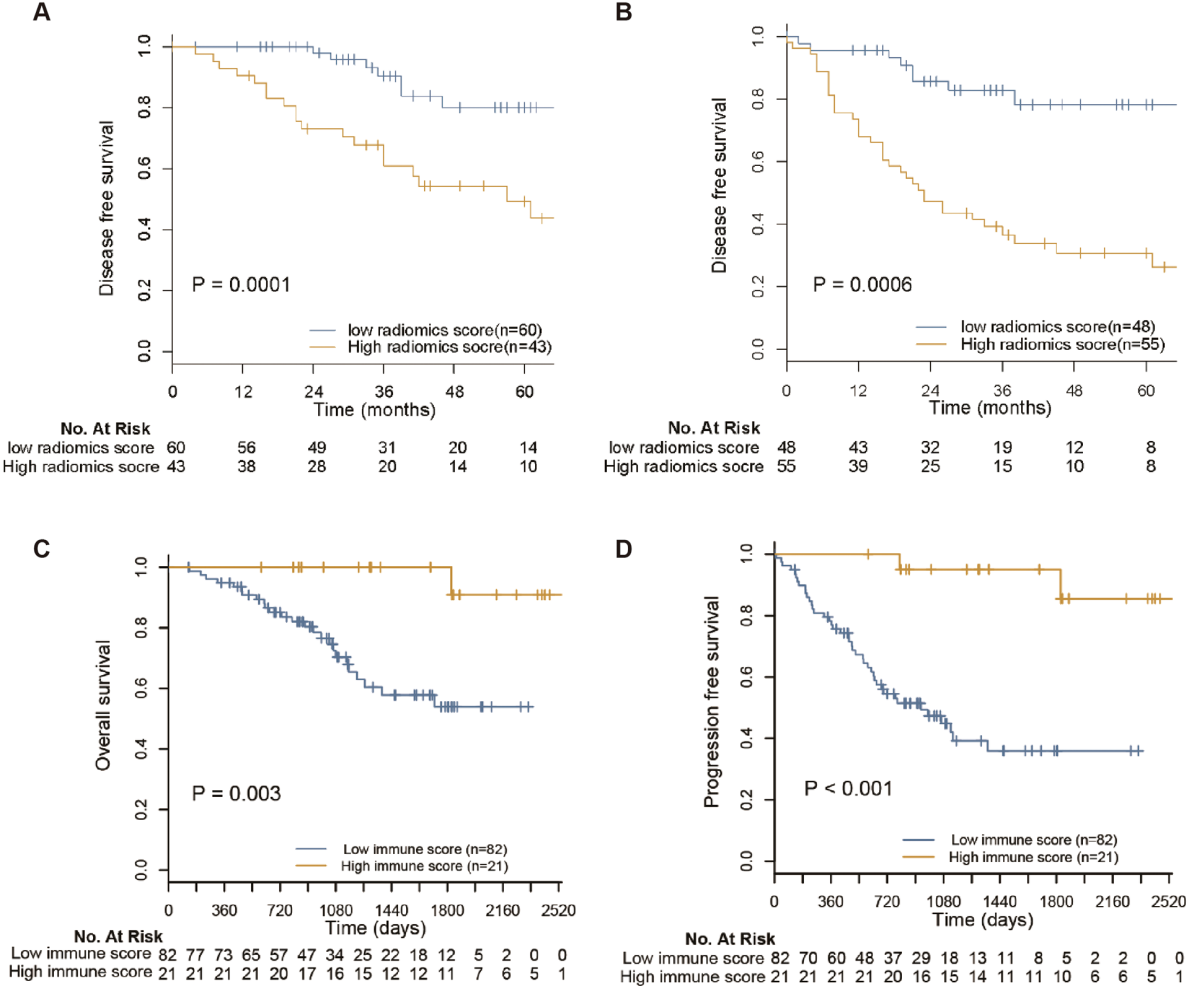
Prognosis value of radiomics score and immune score. The high and low radiomics score subgroups were determined by operating the threshold of 50% to prediction scores of two radiomics models. It showed that the developed two radiomics signatures can divide patients into low and high risk radiomic subtypes with significantly different **(A)** PFS (p=0.0001) and **(B)** OS (p=0.0006). Survival analysis showed that patients with high immune score have notably improved **(C)** OS and **(D)** PFS, confirming immunoscore as a good prognostic factor in mCRC patients.

**Figure 7:**
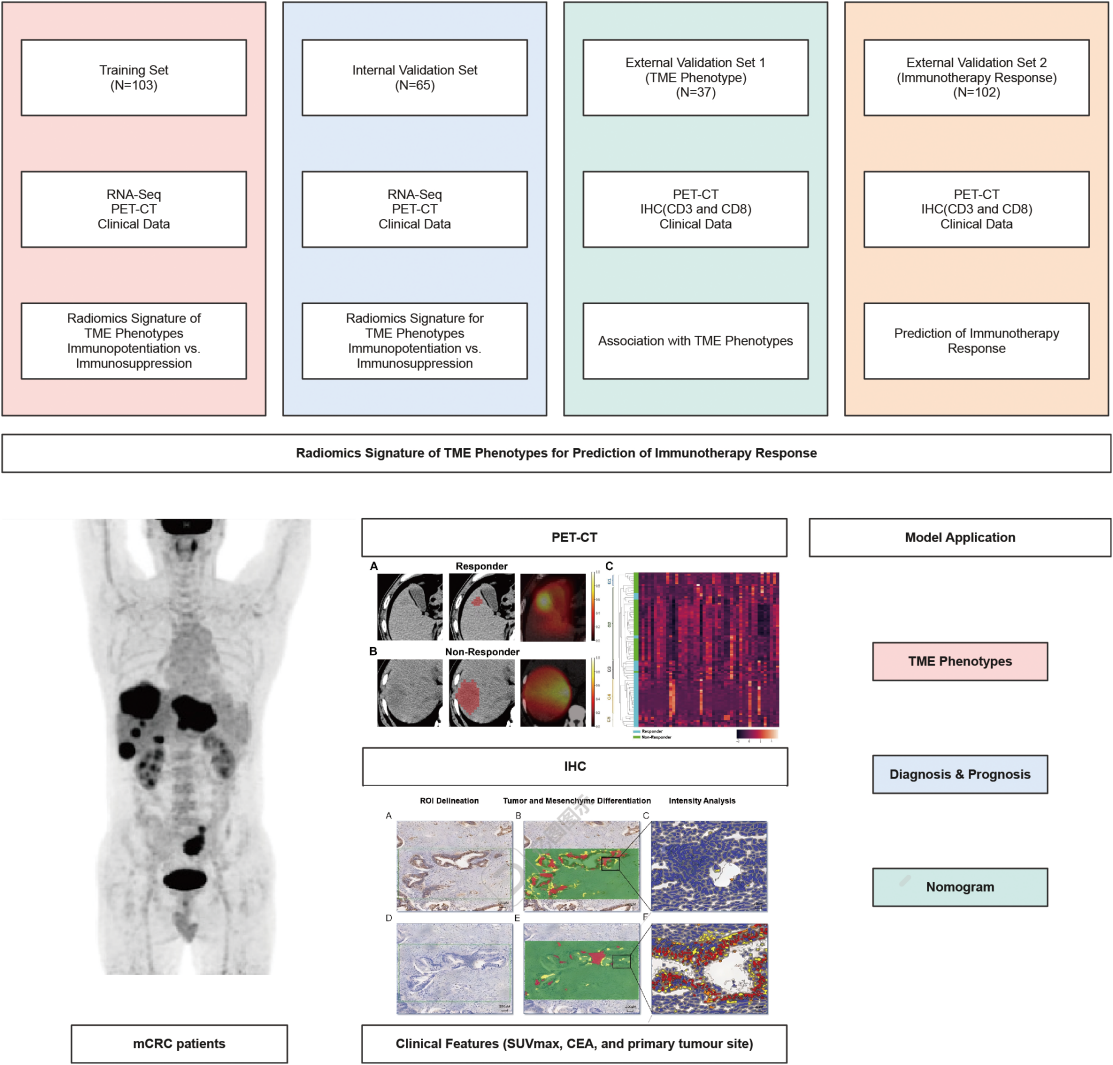
Overall study strategy. The training set consists of 103 mCRC patients whose PET-CT and RNA-seq data are available. The internal validation set (N=65) was utilized to confirm the congruence between the radiomics signature and the TME phenotypes. The TME phenotype cohort (N=37) was employed to reveal the match between the radiomics signature and tumor TME phenotypes. The immunotherapy response cohort (N=102) receiving anti-PD-1/PD-L1 therapy was used to predict the prognostic response to immunotherapy. Baseline PET/CT images, clinical data (SUVmax, CEA, and primary tumour site), and IHC (CD3 and CD8) were retrospectively selected for feature extraction. The radiomics signature for predicting TME phenotypes was developed using random forest algorithm.

The high and low Rad-Score subgroups were determined by applying the 50% threshold of Rad-Score. It was found that Rad-Score could divide mCRC patients into low- and high-risk subgroups, which differed significantly in PFS and OS (both p<0.001) (**Figures 6A, B**). In addition, survival analysis showed that patients with high immunoscore had significantly better PFS and OS, confirming immunoscore as a good prognostic factor in mCRC patients (**Figures 6C, D**). To assess the correlation between Rad-Score and immunoscore, the difference of Rad-Score between the low and high immunoscore groups were compared (**Figure 7**). We found that patients with high immunoscore had significantly lower Rad-Score, confirming the close relationship between radiomics and immune infiltration status.

### Nomograms for the prediction of OS and PFS

**Figure 8:**
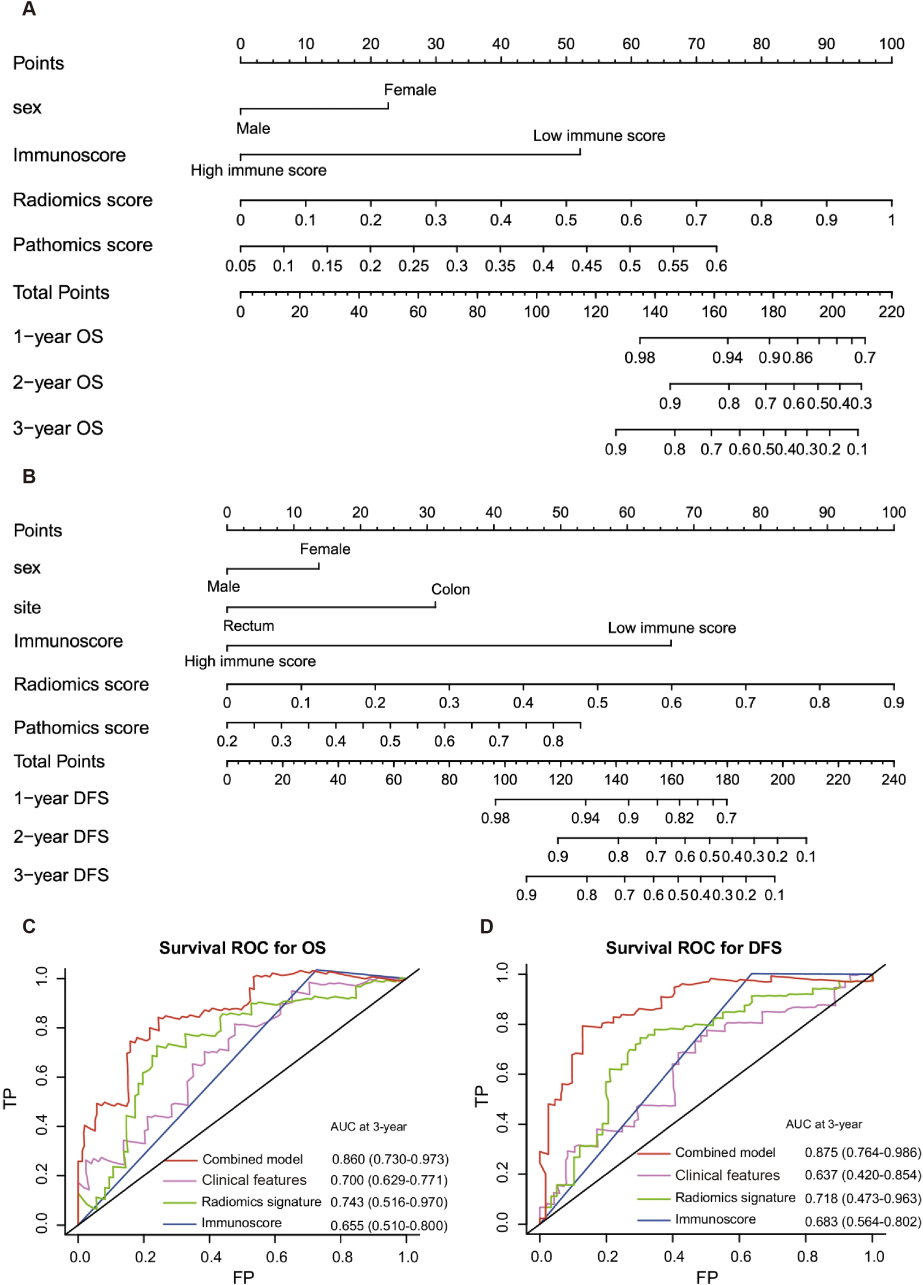
Nomograms for OS and PFS prediction. Combined nomograms incorporating clinical features, radiomics signature and Immunoscore for OS **(A)** and PFS **(B)** prediction. Survival ROC curves demonstrate the prognostic accuracy in predicting OS **(C)** and PFS **(D)** of the combined nomograms.

After multivariate analysis adjusting for clinical variables, sex, Rad-Score and immunoscore remained independent factors for predicting OS and PFS (**Table 2**). Then, two nomograms, combining Rad-Score, immunoscore and clinical factors, were created to predict OS and PFS respectively (**Figures 8A**). The usefulness of combined nomogram was confirmed in the survival ROC analysis for predicting OS (AUC=0.860; **Figure 8**) and PFS (AUC=0.875; **Figure 8**). The calibration curve showed a high accuracy of the combined nomogram model for predicting OS and PFS (**Supplementary Figure S3**). The DCA was then performed to illustrate the clinical decision utility of the combined nomogram (**Supplementary Figure S4**).

**Table 2:** Univariate logistic and cox regression of external validation cohort.

	Number of Events	OS		Number of Events	PFS	
	(relapse/patients)	HR (95% CI)	*P*	(response/patients)	OR (95% CI)	*P*
Sex						
Female	(29/32)	1 (reference)		(11/32)	1 (reference)	
Male	(66/70)	0.36 (0.14-0.96)	0.042	(35/70)	1.91 (0.81-4.66)	0.144
Age, years						
	(64/68)	1 (reference)		(30/68)	1 (reference)	
	(31/34)	0.95 (0.61-1.46)	0.800	(16/34)	1.13 (0.49–2.58)	0.778
Preop. CEA, ng/ml						
	(79/85)	1 (reference)		(41/85)	1 (reference)	
≥200	(16/17)	1.26 (0.74-2.17)	0.395	(5/17)	0.45 (0.13-1.32)	0.161
Preop. CA19-9, U/ml						
	(52/56)	1 (reference)		(27/56)	1 (reference)	
≥200	(43/46)	1.15 (0.76–1.74)	0.499	(19/46)	0.76 (0.34–1.66)	0.486
Clinical risk score^b^						
0-2	(14/15)	1 (reference)		(6/15)	1 (reference)	
3-5	(81/87)	1.05 (0.59–1.86)	0.866	(40/87)	1.28 (0.42–4.10)	0.668
No. of metastases						
	(22/25)	1 (reference)		(9/25)	1 (reference)	
	(73/77)	1.55 (0.95–2.50)	0.077	(37/77)	1.64 (0.66–4.31)	0.295
Maximum size of metastases, cm						
<5	(65/69)	1 (reference)		(32/69)	1 (reference)	
>=5	(30/33)	1.19 (0.75–1.89)	0.452	(14/33)	0.85 (0.36–1.96)	0.708
Radiomics score						
Low	(46/49)	1 (reference)		(10/49)	1 (reference)	
High	(49/53)	0.64 (0.42–0.95)	0.029	(36/53)	8.26 (3.46–21.24)	<0.001
Immune score						
Low	(49/52)	1 (reference)		(9/52)	1 (reference)	
High	(46/50)	0.52 (0.35–0.79)	0.002	(37/50)	13.60 (5.44–37.28)	<0.001

Data presented as %.

CEA, carcinoembryonic antigen; CA19-9, Carbohydrate antigen199; SUV, standardized uptake value; SD, standard deviation.

a All enrolled patients developed synchronous liver metastases. Histology cohort was consisting of patients with colonoscopy biopsy specimens in training and validation cohorts, thus, there was an overlap of patients in three cohorts.

b Clinical risk factors included lymphatic spread of primary cancer, simultaneous metastases, or interval <12 months from primary tumor resection to metastasis, CEA>200 ng/mL, no. of liver metastasis >1, and largest size of liver metastasis > 5 cm. Each risk factor was 1 point.

c Right-sided included tumors from cecal to two thirds of proximal transverse colon; left-sided represented tumors from one third of distal transverse colon to rectum.

## Discussion

In the current study, we presented a PET-CT-based radiomics model that incorporates several novel features to improve the accuracy of predicting response to immunotherapy in mCRC patients. The radiomics model was constructed based on the PET/CT radiomics features extracted via DNNs, IHC biomarkers and clinical features. We believe that this model can not only improve the response rate compared to the current immunotherapy delivery strategy, but also reduce the use of anti-PD-1/PD-L1 in mCRC patients. This study is a step forward in the use of artificial intelligence (AI) for personalized treatment and prognosis prediction in mCRC patients. Further validation, clinical impact and prospective studies are needed to determine the reliability, generalizability and utility of these models for the clinical management of mCRC patients. Taken together, we proposed a promising tool for predicting response to immunotherapy in mCRC patients before treatment, which could help clinicians stratify mCRC patients and identify those who may benefit from immunotherapy.

In the last decade, several radiomics-based models have been proposed to predict response to immunotherapy in various tumors (18, 26–28); however, these methods relied on manually generated ROI labels for image feature extraction, raising the potential that optimal radiomics features for predicting response to immunotherapy may not be involved. Recently, DNNs have been widely used in radiomics research to automatically extract image features. For instance, 256 features (deep features) have been extracted from CT images via a convolutional neural network (CNN) (29). Compared to traditional radiomics models, DNN-based radiomics achieved better performance. Hence, in the current study, CT and PET features were also extracted using DNNs.

There is a consensus that clinical factors can improve the predictive ability of radiomics (30, 31). In this study, clinical factors were also included. SUVmax of PET/CT, pre-operative CEA, and primary tumor site were screened out using the LASSO regression, which have been shown to correlate with immunotherapy efficacy. PD-L1 is the most commonly used biomarker associated with immunotherapy. Nevertheless, PD-L1 measured by IHC staining is not satisfactory as a biological marker for immunotherapy for the majority of tumors (32). Therefore, new biomarkers are needed for predicting and monitoring patient responses to immunotherapy. This study aims to create a PET-CT radiomics signature related to the TME phenotype and immunotherapy efficacy. A radiomics signature of the TME phenotype was derived from PET-CT images and the relationship between the radiomics signature, the TME phenotype, and response to immunotherapy was investigated. As a significant predictor, Rad-Score may be related to the immune activity of the tumor microenvironment. In clinical practice, a high Rad-Score may indicate that a patient is more likely to benefit from immunotherapy. Furthermore, the predicted performance of the radiomics signature was validated in three different cohorts and showed that it is associated with clinical outcomes in mCRC patients treated with immunotherapy.

The immunoenhanced and immunosuppressed phenotypes were examined in this study. We focused on the extent of immune and stromal cell infiltration in TME because lack of immune infiltration is associated with poor immunotherapy response. TME phenotypes are generally divided into immunoenhanced (“ hot tumors “) and immunosuppressive (“ cold tumors “) types, corresponding to tumor microenvironments with abundant and low immune cell infiltration, respectively. Metabolic features in PET images, such as standardized intake value (SUVmax) and metabolic volume, and anatomical features in CT images, such as tumor size, shape, and texture, all reflect tumor heterogeneity. In addition, advanced features extracted by deep learning algorithms can capture the complex heterogeneity of tumors, further improving the ability to identify TME phenotypes. The application of radiomics to TME phenotypic classification has significant advantages, such as non-invasive, high-resolution, and multimodal data fusion capabilities, enabling it to provide comprehensive tumor characterization information based on PET/CT images without relying on invasive tissue biopsies. To better explain the spatial distribution of immune and stromal cells in TME, as we gain a better understanding of mCRC and its immune function in TME, a prospective direction in the future may include possible other immunophenotypes.

In addition, we added CD3 and CD8 IHC staining to the endoscopic biopsy to show the TME phenotype of the primary tumor. The inclusion of these protein markers enables the combined model to further improve the performance of the external validation queue. The Rad-Score is generated by a random forest classifier that screens for features associated with the TME phenotype and is able to distinguish between immune-enhanced and immune-suppressed tumors. In terms of immunotherapy response prediction, radiomics assesses patient response to immunotherapy by building predictive models such as Rad-Score and combination models. The combined model combined Rad-Score, immune score (based on CD3 and CD8 IHC staining results), and clinical features to further improve the accuracy of the prediction of OS and PFS, and the improved performance of the combined model showed that these features were effective. Validation of radiomic models is a key step to ensure their reliability and generalization ability, including internal validation, external validation, and predictive ability validation in immunotherapy response cohorts. Radiomics has demonstrated high accuracy, multimodal data fusion capabilities, and clinical value in immunotherapy response prediction, providing clinicians with a non-invasive tool to evaluate a patient’s potential response to immunotherapy before treatment, thereby optimizing treatment strategies.

There are several limitations in the current study. First, we were not able to include all patients in the study because baseline PET/CT data and tumor tissues were not available, potentially leading to selection bias and confounding. Second, the radiomics model used a semi-automated segmentation pipeline for tumor ROI, which still requires manual initialization and post-processing. Third, the protein markers selected for this study were derived from the literature, and optimal candidates may need to be verified using proteomic data from tumor samples of mCRC patients treated before and after immunotherapy. Finally, due to the retrospective nature of this study, a large prospective study is required to further validate the results.

## Conclusions

In conclusion, we have developed a PET-CT radiomics model for predicting response to immunotherapy in mCRC patients. Our research shows that the PET-CT radiomics model proves to be a promising, non-invasive, cost-effective, and reliable tool for characterizing the TME phenotype and predicting response to immunotherapy in mCRC patients. The robust performance of the radiomics model presented the promise for the development of therapies prior to the administration of conversion therapy administration, with the goal of reducing mortality. Although the results need to be validated in a larger prospective study, they underline the potential for the development of non-invasive biomarkers in the field of immunotherapy.

## Data Availability

The datasets presented in this study can be found in online repositories. The names of the repository/repositories and accession number(s) can be found in the article/**Supplementary Material**.
