# Supplementary material for: A PET-CT radiomics model for immunotherapy response and prognosis prediction in patients with metastatic colorectal cancer
Source: Front Oncol. 2025 May 23;15:1568755. doi: 10.3389/fonc.2025.1568755 (PMC12141312; doi:10.3389/fonc.2025.1568755)
Supplement: Supplementary file 1 [file DataSheet1.docx]

**Supplementary Information**

**Methods**

**Informed consent**

Written informed consent was obtained from all participants prior to inclusion in the study. Patients are informed and consented in the surgery outpatient clinic or, if directly admitted, in the surgical ward, before admission for surgery.

**Study design**

The traning set and internal validation cohort was derived from consecutive mCRC patients of the multi-disciplinary team (MDT) at Shenzhen People’s Hospital, share the same MDT, surgical team, and PET/CT imaging equipment with training cohort, from 01 January 2017 to 31 December 2023 (Figure S1). Inclusion criteria were as follows: (1) Patients were histologically confirmed for colorectal adenocarcinoma with unresectable liver-limited or liver-dominant metastases; (2) PET/CT at baseline were available; and (3) First line treated with with anti-PD-1 monotherapy. Exclusion criteria were as follows: (1) Resectable liver metastases; (2) Wide-type KRAS/NRAS; (3) No measurable liver metastasis; (4) No efficacy assessment; and (5) No follow-up information. The external validation cohort came from the MDT of the Tenth Affiliated Hospital of Southern Medical University and Shenzhen Pingshan District People’s Hospital, from 01 January 2018 to 31 December 2023, and these patients come from different geographical regions and were subjected to diverse PET/CT imaging equipment, with the data batch effect being calibrated by the respective doctors at their respective hospitals. Additional exclusion criteria compared to the Shenzhen People’s Hospital cohort included no primary tumour colonoscopy slides before treatment. This study was approved by the Ethics Committee of Shenzhen People’s Hospital, the Tenth Affiliated Hospital of Southern Medical University and Shenzhen Pingshan District People’s Hospital, in accordance with the Declaration of Helsinki. All study participants have provided informed consent.

The immune phenotype dataset consisted of 42 patients, who had the two most extreme tumour immune phenotypes: immune-inflamed or immune-desert, irrespective of treatment delivered. In the absence of pathological analysis of the tumour immune infiltration (because of the retrospective nature of this study), tumours that were defined as inflamed either had recognised sensitivity to immunotherapy. Immune-desert tumours were defined as those that were known to have poor lymphocyte infiltration. This dataset was used to evaluate the concordance of the radiomic-signature with tumour immune phenotype of tumours. In the external validation set, PET-CT and IHC (CD3 and CD8) data were available, allowing estimation of the quantity of CD3 and CD8 cells and radiomic analysis of the corresponding images of the tumour biopsy samples. More specifically, the gene expression signature from RNA-seq was used to estimate the abundance of CD3 and CD8 cells. The genomic data were then aligned with the images to generate the radiomic signatures.

The immunotherapy-treated cohort consisted of 176 patients with advanced CRC enrolled in anti-PD-1 monotherapy at the Tenth Affiliated Hospital of Southern Medical University. This cohort was used to infer the association of the radiomic signature with clinical response in accordance with the Response Evaluation Criteria in Solid Tumors (RECIST) version 1.1, progression-free survival, and overall survival.

**Frozen fresh tumour tissue and normal sample**

Before formalin fixation and immediate upon retrieval of the specimen, representative fresh tumour samples are obtained (at least three per tumour), stored in meticulously marked vials, and frozen in liquid nitrogen. Time is kept to a minimum between resection and delivery at Pathology in order to minimize loss of RNA quality, usually delivered by an orderly within 15 min of retrieval from the operating room to the laboratory.

For the rare occasional procedures commencing or proceeding outside opening hours of the Department of Pathology, the surgeon in charge samples the fresh tumour biopsies per protocol and provides this in a portable insulated box container with dry ice for storage, normally <12 h. Samples are then collected by a technician and processed per protocol as early as possible the next morning.

**RNA extraction**

RNA was extracted from freshly frozen tumour and normal (surgical resection margins or normal tissue sampled distant from primary tumour) using the QIACUBE (Qiagen) instrument and dedicated reagents and kits, according to manufacturers instructions. Weighted 15-20 mg of tissue are resuspended in lysis buffer and homogenized in the presence of 5 mm Ø steel beads, in a TissueLyser LT (Qiagen), at 50 Hz, for 4 min. Then, the QIACUBE instrument was used to extract RNA via the use of AllPrep RNA Universal Kit. Concentration, purity (A260/280) and presence of phenol and protein contaminants in the eluted sample (A260/230) are measured and noted with a NanoDrop (ThermoFischer) intrument. Extracted RNA was labelled and stored at -80 °C in the aforementioned intramural biobanking facility.

**RNA-seq**

The TruSeq RNA Access target enrichment and library preparation protocols were performed using 250 ng of template RNA. Sequencing was done using synthesis chemistry targeting 50 M reads with a read length of 2x100 bp per sample on a HiSeq 2500 instrument. Transcripts with low counts across the samples (>30% zeros) were excluded, leaving 21,430 transcripts for analysis. Upper quartile normalization was applied to render gene expression values comparable among different samples. Tumors with low gene expression across the genome (>50% genes with zero counts) were removed. Data were log2-transformed prior to downstream analysis.

To deconvolve immune cell phenotypes from the RNA-seq data, CIBERSORTx was applied based on the validated leukocyte gene signature matrix. The variation of each phenotype across the samples (standard deviation >0.1) was the criterion to define an immune feature as represented in our dataset. As a result, 9 hematopoietic cell phenotypes were selected based upon representation in the dataset: naive and memory B cells, plasma cells, CD8+ T cells, resting and activated memory CD4^+^ T cells, M0 and M2 macrophages, and activated mast cells.

**Regions of interest (ROI) outlining**

All patients were asked to fast at least 6 h (until reaching a serum glucose level of <150 mg/dL) and was injected with approximately 3.7 MBq/kg body weight of 18F-FDG. 18F-FDG PET/CT scans were performed using a uMI 510/uMI 780 scanner (United Imaging Healthcare, Shanghai, China). Three dimensions images were obtained approximately 60 min after the 18F-FDG injection, from the skull base to the mid-thigh. The PET images were reconstructed onto a 128 × 128 matrix with CT-based attenuation using the ordered subset expectation maximisation algorithm. The diagnostic CT scan parameters were as follows: Discovery VCT (140 mAs; 120 kV; pitch, 0.516; slice thickness, 1.25 mm), uMI 510/uMI 550/uMI 780 (140 mAs; 120 kV; pitch, 0.9875; slice thickness, 1.0 mm), or Gemini TF 64 (150 mAs; 120 kV; pitch, 0.83; slice thickness, 5.0 mm).

PET images were segmented using a uWS-MI workstation (United Imaging Healthcare, Shanghai, China). The regions of interest (ROI) of PET images were manually enclosed in a cropping box. This segmentation method combines the threshold algorithm and the region-growing algorithm. The former was determined with lesion segmentation through iteration with the seed pixels and those pixels with intensity values greater than the optimal to generate the lesion region. Compared to the frequently employed relative or absolute fixed SUV thresholds, this method utilises all the information from the signal to the background, resulting in superior lesion delineation. Moreover, it is independent of image properties, scanner types, reconstruction, and imaging noise. The CT image ROIs were delineated according to their corresponding ROIs in the PET images.

All PET/CT images were resampled to be 256 × 256 pixels (x-y axes), so that each modality share a same coordinate space. PET images were normalized through a transformation into standard uptake value (SUV) to account for the difference in patient mass and the amount of tracer administered. To get a balanced class proportion for network training, we only kept PET slices which contain delineated lesions. We also conducted a dilation operation using a (10,10) kernel on the segmented ROI to keep more information near lesion boundaries that can retain relevant information about tumor microenvironment. Specifically, we selected three most relevant CT slices for each PET slice overlapping with the tumor ROI.

We extracted features for each area of interest, i.e., the intratumoral and peritumoral regions, using Python, pyradiomics. A final collection of 479 quantitative features, including 14 shape features, 90 first-order intensity features, and 375 s-order and higher-order texture features, was extracted. By evaluating all radiomics features extracted using intra- and inter-class correlation coefficients (ICC), we assess inter- and intra-observer consistency and repeatability. We selected features with an ICC greater than 0.75 ensured the reliability of extracted.

**Feature Extraction of PET/CT images**

*Preparation details:* All PET/CT images were resampled to be 256 × 256 pixels (x-y axes), so that each modality share a same coordinate space. PET images were normalized through a transformation into standard uptake value (SUV) to account for the difference in patient mass and the amount of tracer administered. To get a balanced class proportion for network training, we only kept PET slices which contain delineated lesions. We also conducted a dilation operation using a (10,10) kernel on the segmented ROI to keep more information near lesion boundaries that can retain relevant information about tumor microenvironment. Specifically, we selected three most relevant CT slices for each PET slice overlapping with the tumor ROI. This number can be adjusted according to the imaging slice thickness and slice number ratios between each modality, and we empirically found three worked well on our data.

Radiomics features can be affected by equipment and acquisition conditions, therefore we preprocessed the obtained DICOM images using the following steps: (i) Z-score normalization, where we normalize the intensity of all PET-CT images using the Z-score calculation (Caret package); (ii) Resampling, where we used Simple ITK software to standardize the voxel spacing to 1.0×1.0×1.0 mm^3^.

*Feature Extraction details:* For each PET/CT image pair with one PET slice and three CT slices, the PET feature extraction was conducted in basic 2D, while the CT features were extracted in a 2.5D manner using $1\times1$ convolution. The network backbones were Resnet-34 for CT and EfficientNet-b3 for PET. We used the ReLU and Sigmoid Linear Unit (SiLU) as the activation function for Resnet-34 and EfficientNet-b3, respectively. We used the pytorch official implementation of these backbones (<https://github.com/pytorch/vision/blob/main/torchvision/models/efficientnet.py> and <https://github.com/pytorch/vision/blob/main/torchvision/models/resnet.py>).

Specifically, the Resnet-34 architecture consists of 1 input layer, 1 convolutional layer with 64 filters and a stride of 2, 1 batch normalization, 1 ReLU activation, 1 maxpooling, 8 repeated sequential blocks ,1 average pooling layer, 1 linear layer. Each sequential block contains 4 repeated basic blocks, each which consist of 2 convolutional layers, 2 batch normalization layers and 2 Relu layers. The EfficientNet-b3 architecture consists of 1 input layer: 2 Conv2dNormActivation blocks, 26 MBConv blocks, a drop-out layer and a softmax layer.

*Network training details*: We trained the networks to build a relation between PET/CT images and bevacizumab efficacy. We used binary cross entropy as the loss function and the learning rate of Adam optimizer learning rate was set as 0.0001 initially. The batch size was 64. The learning rate would be automatically reduced by a factor of 5 when loss didn’t improve over 30 epochs. We used different augmentation techniques including horizontal/vertical-flip, random rotation ($-30^{\circ} to 30^{\circ}$), random zoom to control overfitting. The training was stopped after the validation loss didn’t improve over 50 additional epochs.

*Gaussian mixture model transformation details:* Specifically, we encoded the distribution of CNN features from different layers using the GMM parameters $(\omega, \mu, \sigma^{2})$.

Formally, let $z_{l,i}$ be the $i$th feature map of layer in the network. We model the distribution $p(z_{l,i})$ as a weighted sum of $k$ Gaussian components, i.e.,

$$p\left( Z_{l},i \right)=\sum_{j=1}^{k} \omega_{l,i}^{j}\mathcal{G(}Z_{l,i};\mu_{l,i}^{j},\left( \sigma_{l,i}^{j} \right)^{2})$$

where$\omega^{j}$is the mixture weight of the $j$th component, and $\mathcal{G}$ is the Gaussian probability distribution function with mean $\mu^{j}$and variance$\left( \sigma^{j} \right)^{2}$ defined as

$$\mathcal{G} \left( x;\mu,\sigma^{2} \right)=\frac{1}{\sigma\sqrt{2\pi}}exp(-\frac{1}{2\sigma^{2}}\left( x-\mu\right)^{2})$$

Using this model, we represent feature map $z_{l,i}$ by a row vector with $3 \times k$ elements:

$$y_{l,i}=[\mu_{l,i}^{\left( 1 \right)},\sigma_{l,i}^{\left( 1 \right)},\omega_{l,i}^{\left( 1 \right)},\ldots,\mu_{l,i}^{\left( k \right)},\sigma_{l,i}^{\left( k \right)},\omega_{l,i}^{\left( k \right)}]$$

Where $m_{l}$ is the number of feature maps in layer $l\in\{1, ... , n\}$. Considering feature maps in each convolutional layer and in the first fully connected layer, we arrived at a feature descriptor $y_{GMM-CNN}$ with a total of $3\times k \times\sum_{l=1}^{n} m_{l}$ features, where n is the total number of layer and $m_{l}$ is the number of feature maps in layer $l$

$$y_{GMM-CNN}=[y_{1,1}\ldots y_{1,m_{1}}\ldots y_{n,1}\ldots y_{n,m_{n}}]$$

This feature descriptor $y_{GMM-CNN}$, is the output of the GMM to be served as the image feature.

**Clinical feature selection**

Recognizing the significant collinearity among the PET parameters, which indicated varying degrees of correlation among multiple variables (Figure S2), we adopted a two-stage method to address this issue. Initially, we applied LASSO regression directly to all variables instead of performing traditional univariate analysis. LASSO regression reduces complexity and mitigates multicollinearity by penalizing the absolute size of the regression coefficients, compressing some coefficients and setting others to zero, effectively selecting more relevant variables (Figure S3). Subsequently, the variables that remained significant were further analyzed through stepwise regression, specifically a bidirectional approach that iteratively added and removed predictors to find the optimal model. This combination of lasso followed by stepwise regression allows for more robust feature selection in the presence of multicollinearity. After the variable selection process, four variables were used to construct the predictive model: SUVmax (OR =1.265, 95% CI: 1.144-1.420, P<0.001), metabolic tumor volume (OR =0.976, 95%CI: 0.953-0.996, P=0.02), pathology type (OR =7.301, 95%CI: 1.876-38.682, P=0.008), and CA125 level (OR =6.736, 95%CI: 2.594-19.242, P<0.001).

**Immunoscore**

Immune response was calculated based on mean densities of CD3^+^ and CD8^+^ in tumor center and invasive margin in all of the patients in the study. The calculated mean density was used to divide the individual cases into “high” or “low” immune response. Cases with mean density ≥ 75-percentile were regarded as “high” immune response. Patients were stratified from I0 to I4 according to the “Immunoscore”, based on the total number of observed high densities (CD3^+^ and CD8^+^ TILs). The final immune score was categorized based on mean percentiles for all four markers, and divided into immune score “low”, “intermediate” and “high” based on the number of markers (0-4) ≥ 75th percentile.

**Pathomics score**

CD3- and CD8-stained slides were scanned at 40 × magnification using Leica SCN400 slide scanner (Leica Microsystems, Wetzlar, Germany) and uploaded to image analysis software, Visiopharm® (Hoersholm, Denmark). The region of tumor center and invasive margin were marked manually on whole slides in Visiopharm® and these regions (region of interest, ROI) were used for CD3+ and CD8+ cell quantification. Visiopharm® identified and measured the area of positive cells using digital image analysis. The area of positive cells were transformed into number of positive tumor-infiltrating lymphocytes (TILs) based on the estimation of mean area of a lymphocyte. The number of positive CD3^+^ and CD8^+^ TILs was calculated per square millimeters (mm^2^), further represented as pathomics score.

**Supplementary Information 1**


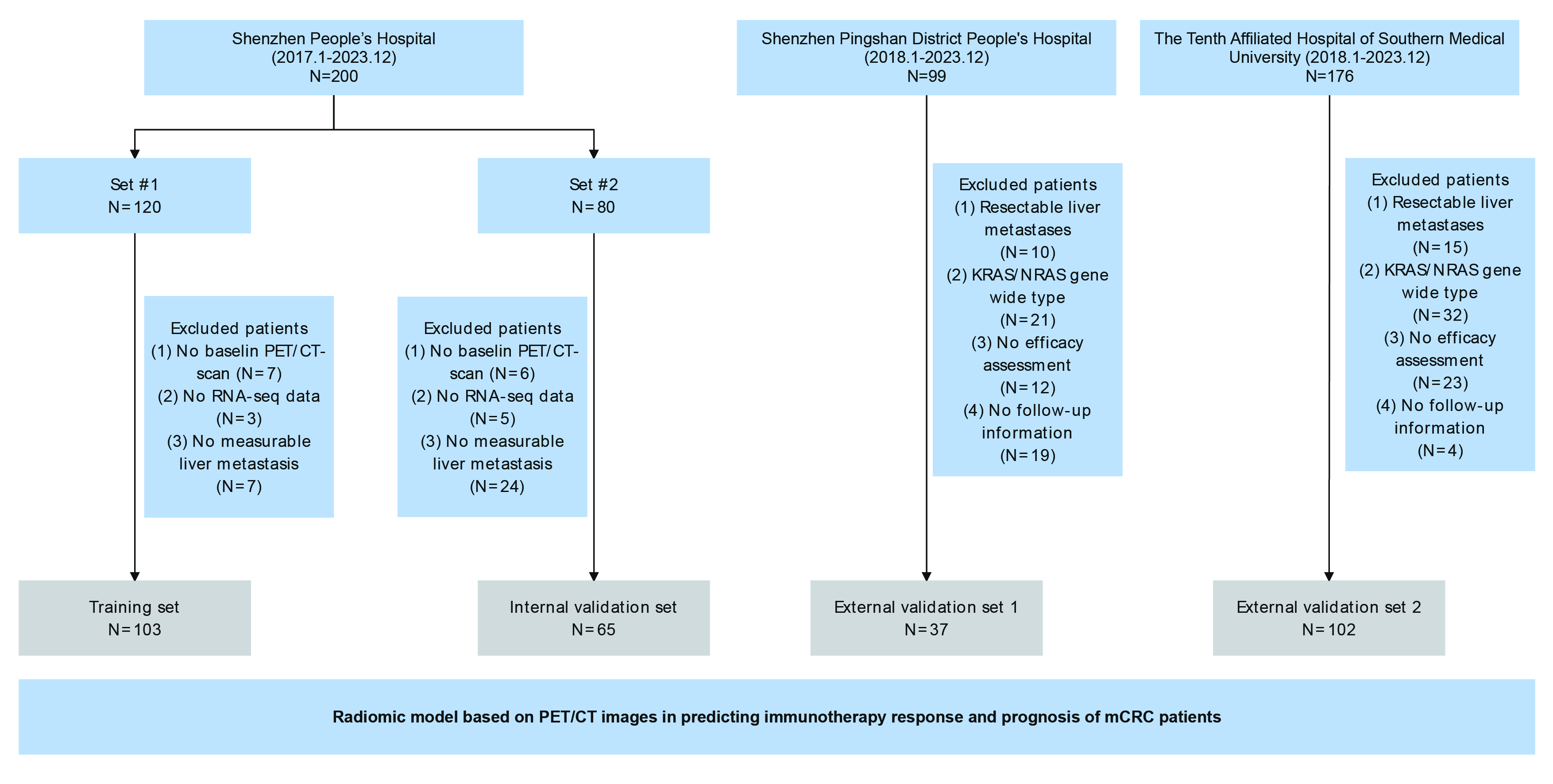


**Figure S1. Study design and participants.**


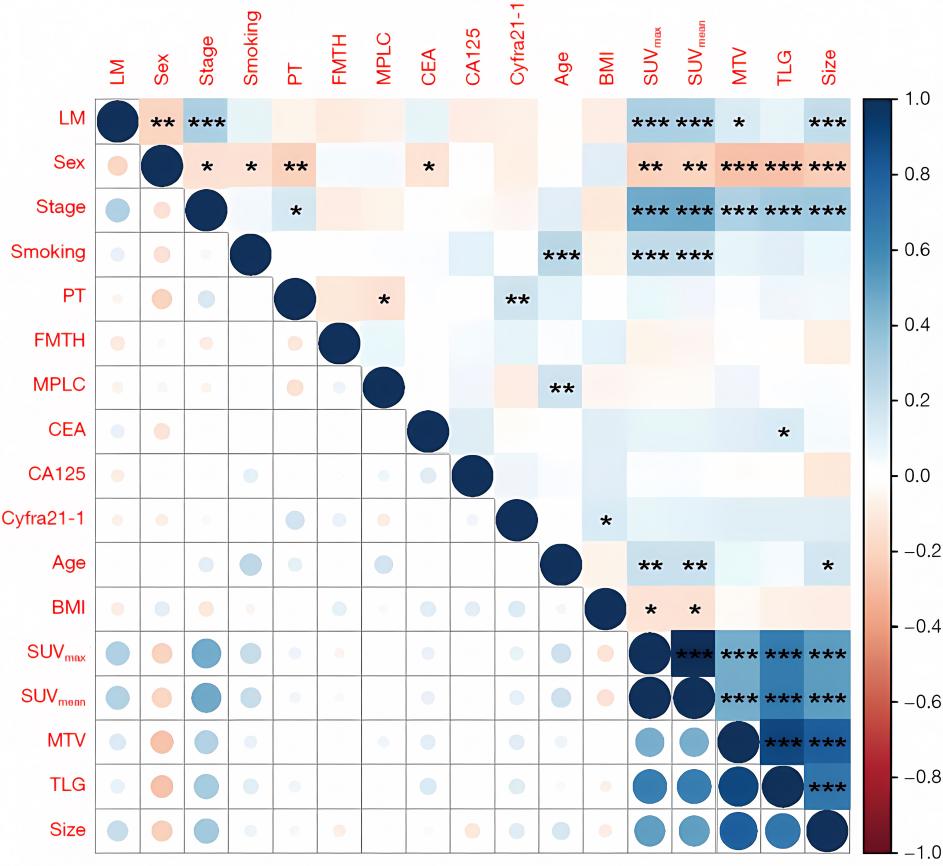


**Figure S2. Correlation heatmap showing the correlation between various factors.** *, P<0.05; **, P<0.01; ***, P<0.001. LM, lymphatic metastasis; PT, pathology type; FMTH, family malignant tumor history; MPLC, multiple primary lung cancer; BMI, body mass index; CEA, carcinoembryonic antigen; CA125, carbohydrate antigen 125; Cyfra21-1, cytokeratin 19 fragment; SUVmax, maximum standardized uptake value; SUVmean, mean standardized uptake value; MTV, metabolic tumor volume; TLG, total lesion glycolysis.


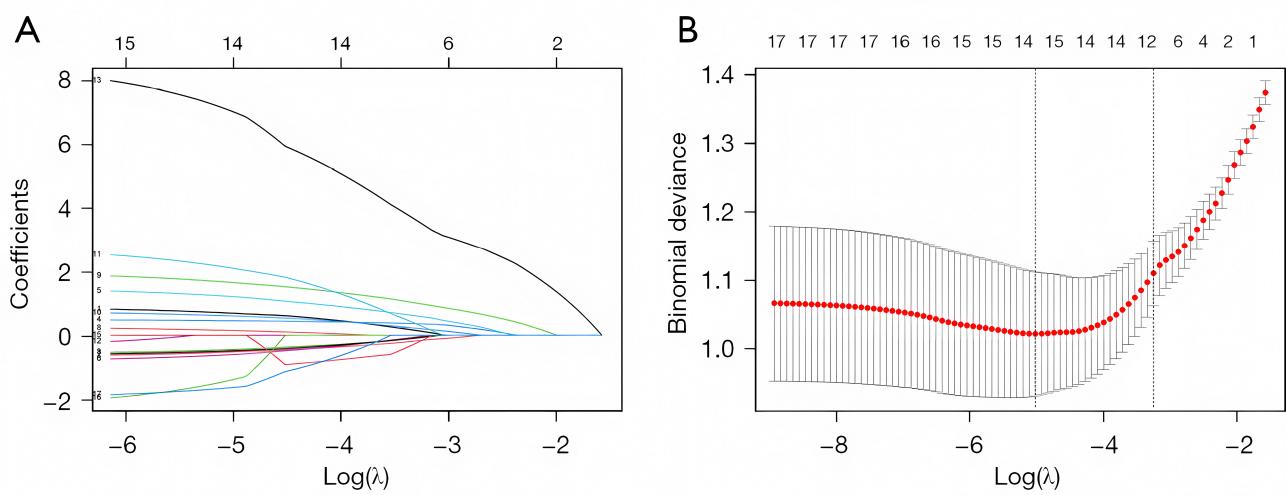


**Figure S3. Least absolute shrinkage and selection operator (LASSO) regression to all 17 variables.**


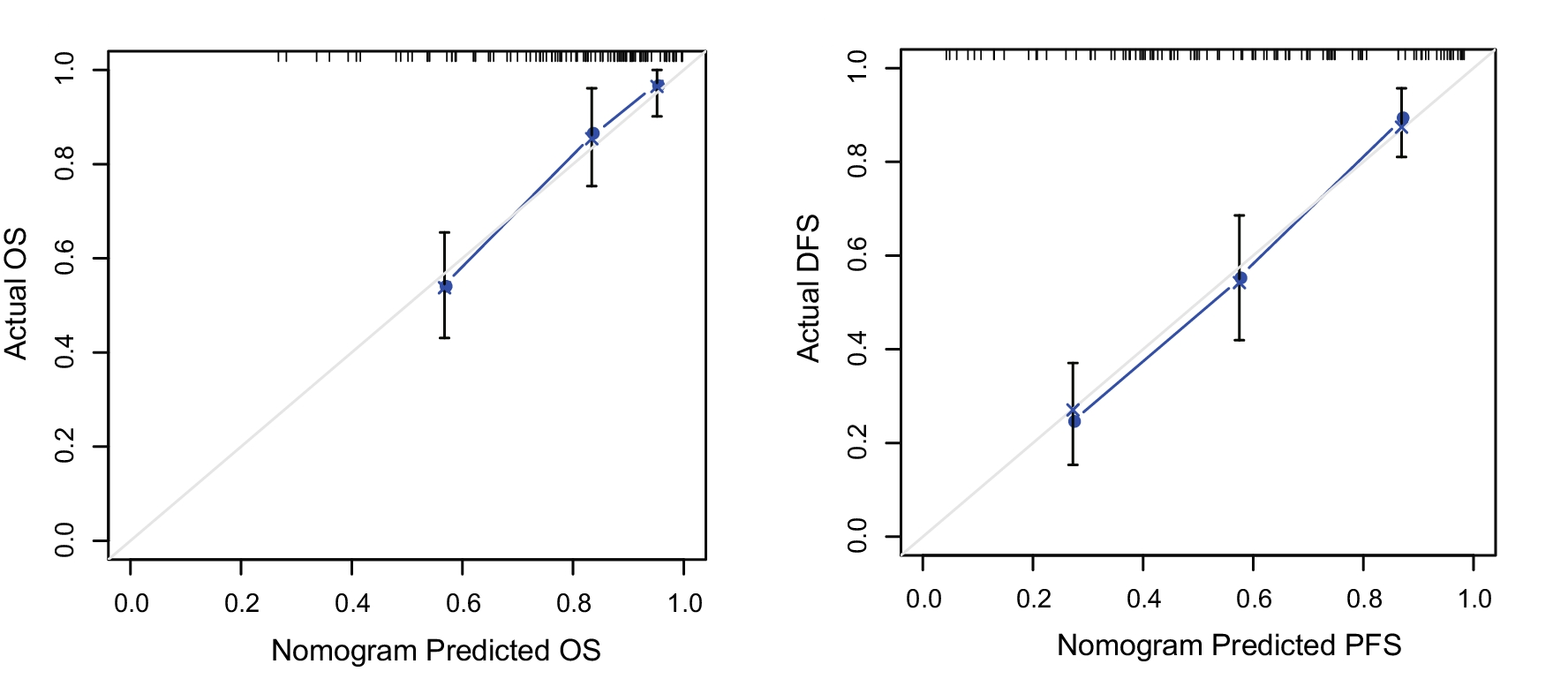


**Figure S4. Calibration curves of combined nomograms for OS and DFS prediction.**

**
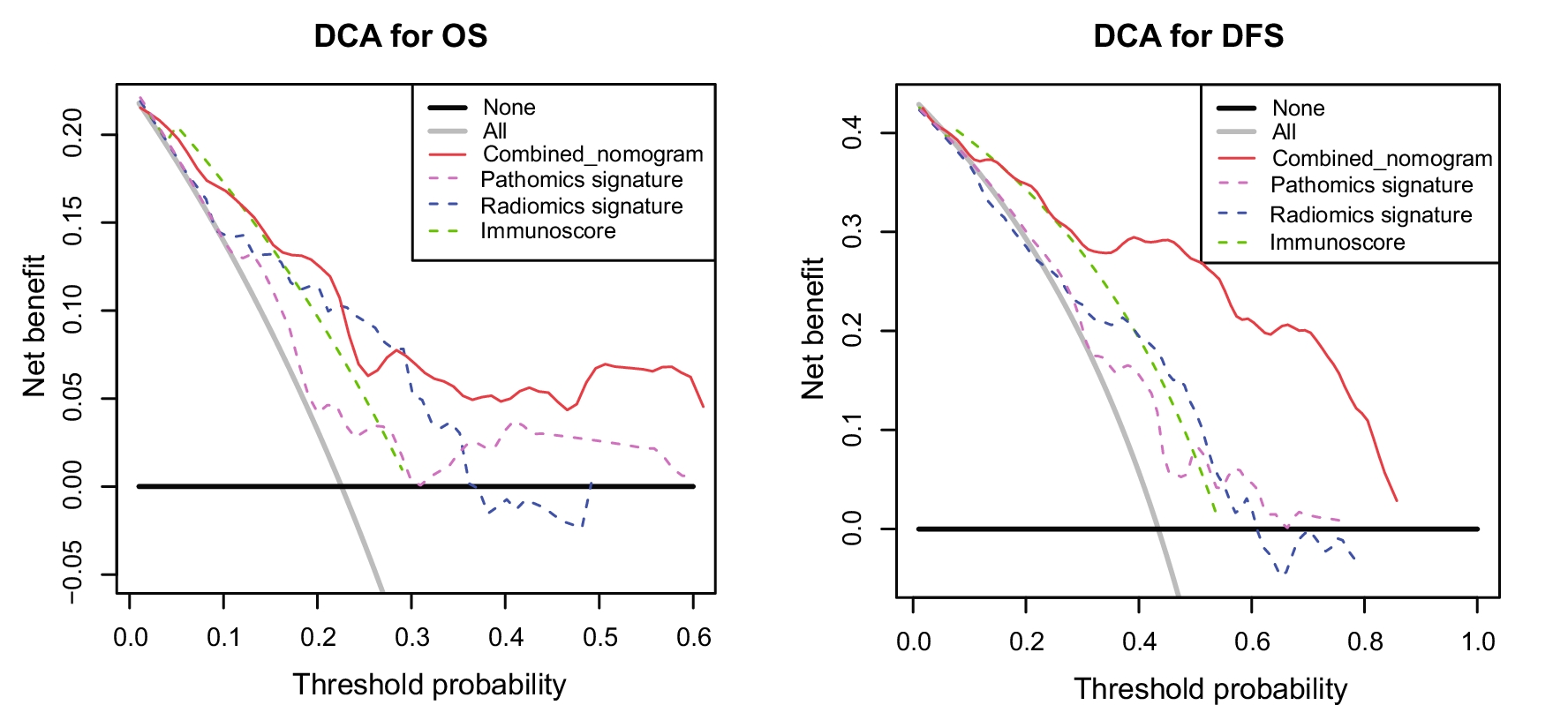
**

**Figure S5. Decision curve analysis demonstrate the clinical utility in predicting OS and DFS of the combined nomograms, radiomics signature and Immunoscore.**
